# Supplementary figures and images for: Ankyrin domains across the Tree of Life
Source: PeerJ. 2014 Feb 6;2:e264. doi: 10.7717/peerj.264 (PMC3932732; doi:10.7717/peerj.264)

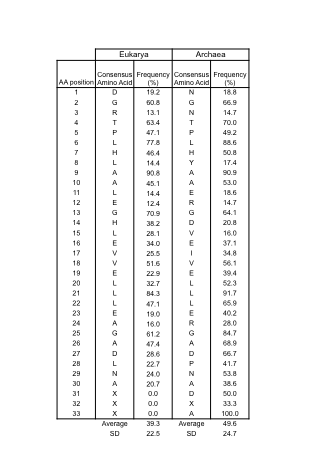

Supplement: Supplemental Information 4 [file peerj-02-264-s004.png]

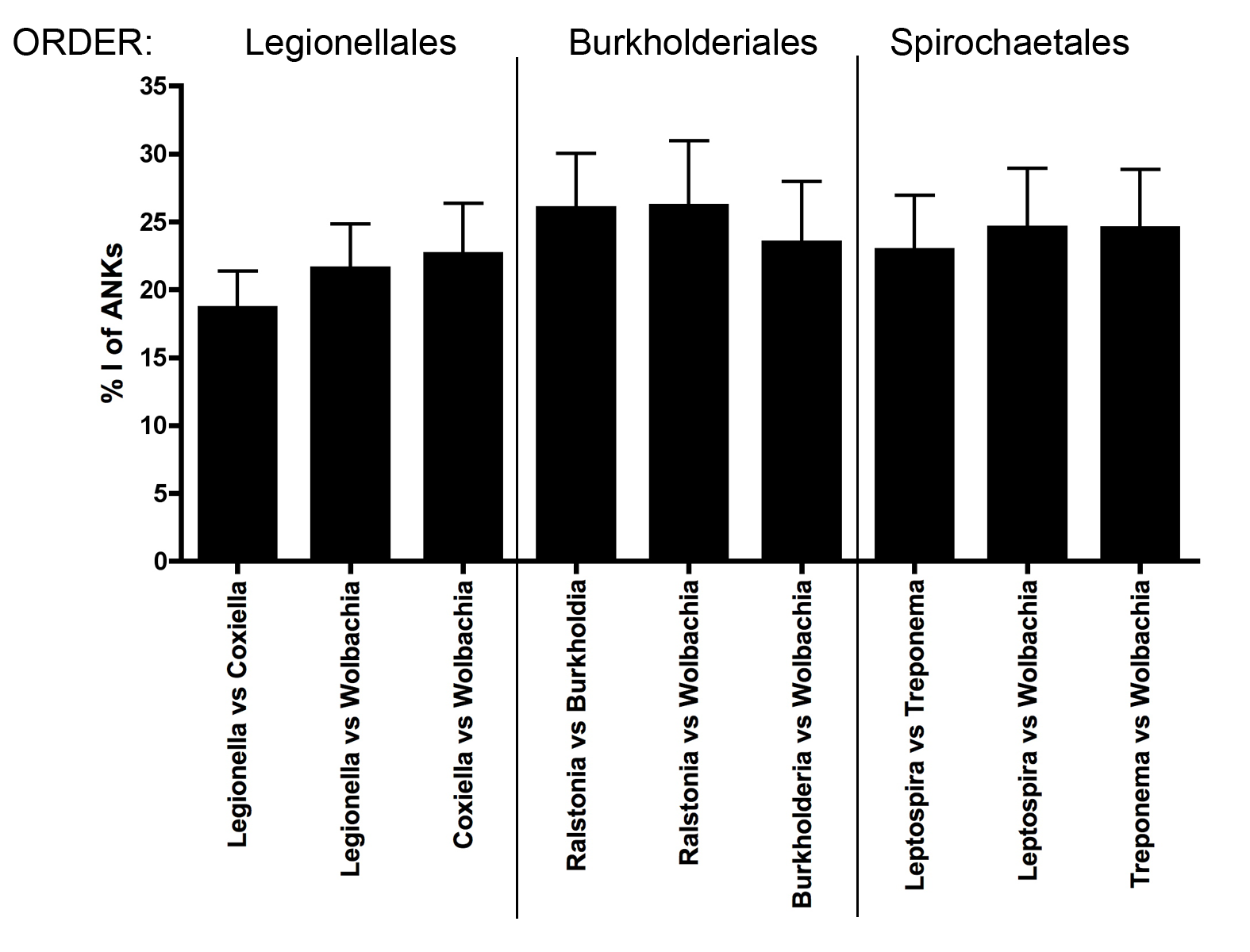

Supplement: Supplemental Information 9 — All ANK repeat sequences from the following species were analyzed: Legionella pneumophila Philadelphia 1, Coxiella burnetii Dugway 7E9-12, Wolbachia pipientis wMel, Ralstonia solanacearum PSI07, Burkholderia vietnamiensis G4, Leptospira biflexa serovar Patoc strain ’Patoc 1 (Paris), Treponema pallidum pallidum Nichols. To analyze the individual ANK repeats, the ANK-containing proteins sequence ID was obtained from SUPERFAMILY v1.75 and the amino acid sequence information was obtained from NCBIs Proteins database (http://www.ncbi.nlm.nih.gov/protein/). SMART (http://smart.embl-heidelberg.de/) was used to identify the number and location of each individual ANK repeat in the ANK-containing protein. For the amino acid sequence identity analysis, individual ANK repeat sequences were aligned using MUSCLE and the percent identity of the sequences was calculated in Geneious Pro 5.6.2. When comparing ANK repeat sequences from two strains, the average of all combinations of ANK repeat comparisons was used. All species ANK repeat sequences were compared to Wolbachia pipientis wMel to show that the level of identity was the same between species of the same order, and that of a different order (Wolbachia). [file peerj-02-264-s009.png]

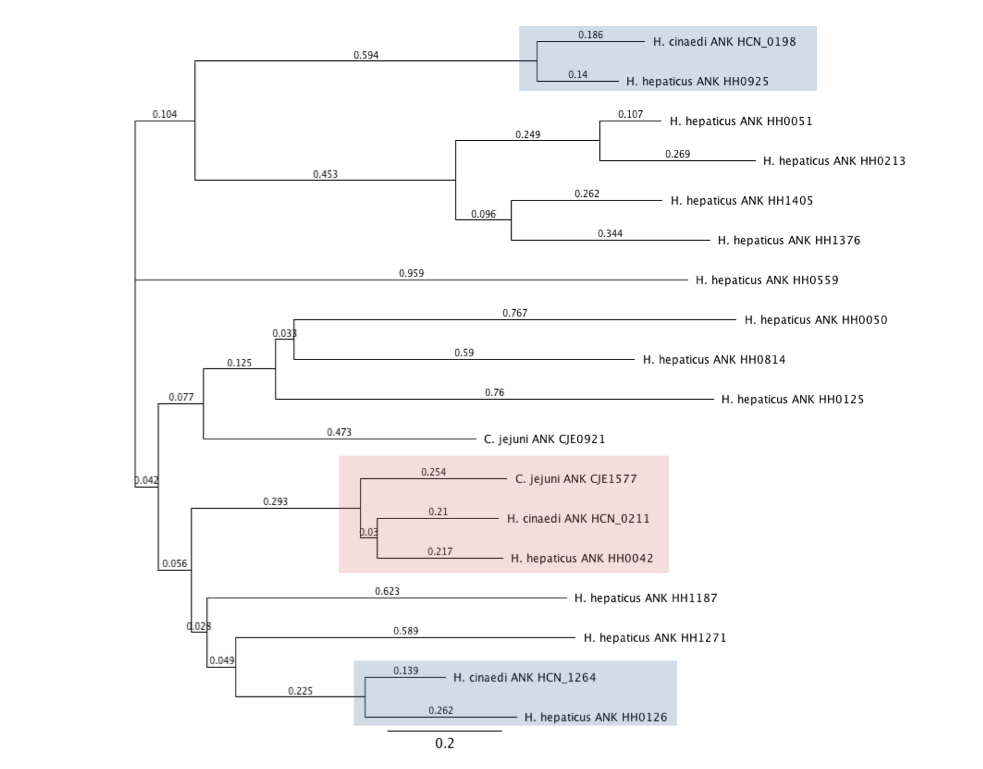

Supplement: Supplemental Information 13 — SMART was used to identify ANK repeats, and the ANK domain contained all ANK repeats and some internal linker sequences. MUSCLE alignment was used to align amino acid sequences. INDELS and ends of amino acid sequences were removed after being aligned. Geneious builder was used to build the Neighbor-Joining tree. Blue boxes; Helicobacter hepaticus & Helicobacter cidaedi orthologs Red Box: Helicobacter hepaticus, Helicobacter cidaedi, and C. jejuni orthologs. [file peerj-02-264-s013.png]
