# Supplementary material for: Ankyrin domains across the Tree of Life
Source: PeerJ. 2014 Feb 6;2:e264. doi: 10.7717/peerj.264 (PMC3932732; doi:10.7717/peerj.264)
Supplement: Supplemental Information 5 [file peerj-02-264-s005.doc]

| **Species** | **Lifestyle** | **Ankryin proteins** | **Total Protein Number** | **% Proteins with Ank** |
| --- | --- | --- | --- | --- |
| Acidobacteria bacterium Ellin345 (Candidatus Koribacter versatilis Ellin345) | Extracellular | 4 | [4777.00](http://www.ncbi.nlm.nih.gov/genome/proteins/1384?project_id=58479) | 0.0837 |
| Acinetobacter baumannii 1656-2 | Extracellular | 4 | [3824.00](http://www.ncbi.nlm.nih.gov/genome/proteins/403?project_id=158677) | 0.1046 |
| Acinetobacter baumannii ACICU | Extracellular | 4 | [3759.00](http://www.ncbi.nlm.nih.gov/genome/proteins/403?project_id=58765) | 0.1064 |
| Acinetobacter baumannii ATCC 17978 | Extracellular | 4 | [3367.00](http://www.ncbi.nlm.nih.gov/genome/proteins/403?project_id=58731) | 0.1188 |
| Acinetobacter baumannii ATCC 19606 | Extracellular | 4 | [3766.00](http://www.ncbi.nlm.nih.gov/genome/proteins/403?project_id=40853) | 0.1062 |
| Acinetobacter baumannii AB307-0294 | Extracellular | 4 | 3451.00 | 0.1159 |
| Acinetobacter baumannii SDF | Extracellular | 4 | 2975.00 | 0.1345 |
| Acinetobacter calcoaceticus PHEA-2 | Extracellular | 4 | [3599.00](http://www.ncbi.nlm.nih.gov/genome/proteins/2267?project_id=83123) | 0.1111 |
| Actinomyces graevenitzii C83 | Extracellular | 4 | [1853.00](http://www.ncbi.nlm.nih.gov/genome/proteins/2204?project_id=80681) | 0.2159 |
| Agrobacterium radiobacter K84 | Extracellular | 4 | [6684.00](http://www.ncbi.nlm.nih.gov/genome/proteins/177?project_id=58269) | 0.0598 |
| Anabaena variabilis ATCC 29413 | Extracellular | 4 | [5710.00](http://www.ncbi.nlm.nih.gov/genome/proteins/1648?project_id=58043) | 0.0701 |
| Arcobacter sp. L | Extracellular | 5 | [2848.00](http://www.ncbi.nlm.nih.gov/genome/proteins/13574?project_id=158135) | 0.1756 |
| Bacillus anthracis Ames | Extracellular | 4 | [5328.00](http://www.ncbi.nlm.nih.gov/genome/proteins/181?project_id=57909) | 0.0751 |
| Bacillus anthracis Ames Ancestor | Extracellular | 4 | [5484.00](http://www.ncbi.nlm.nih.gov/genome/proteins/181?project_id=58083) | 0.0729 |
| Bacillus anthracis Sterne | Extracellular | 4 | [5289.00](http://www.ncbi.nlm.nih.gov/genome/proteins/181?project_id=58091) | 0.0756 |
| Bacillus anthracis str. A0248 | Extracellular | 4 | [5292.00](http://www.ncbi.nlm.nih.gov/genome/proteins/181?project_id=59385) | 0.0756 |
| Bacillus anthracis str. CDC 684 | Extracellular | 4 | [5902.00](http://www.ncbi.nlm.nih.gov/genome/proteins/181?project_id=59303) | 0.0678 |
| Bacillus anthracis str. H9401 | Extracellular | 4 | [5791.00](http://www.ncbi.nlm.nih.gov/genome/proteins/181?project_id=162021) | 0.0691 |
| Bacillus cereus 03BB102 | Extracellular | 4 | [5606.00](http://www.ncbi.nlm.nih.gov/genome/proteins/157?project_id=59299) | 0.0714 |
| Bacillus cereus ATCC14579 | Extracellular | 4 | [5255.00](http://www.ncbi.nlm.nih.gov/genome/proteins/157?project_id=57975) | 0.0761 |
| Bacillus cereus B4264 | Extracellular | 4 | [5398.00](http://www.ncbi.nlm.nih.gov/genome/proteins/157?project_id=58757) | 0.0741 |
| Bacillus cereus E33L | Extracellular | 5 | [5641.00](http://www.ncbi.nlm.nih.gov/genome/proteins/157?project_id=58103) | 0.0886 |
| Bacillus cereus G9842 | Extracellular | 4 | [5857.00](http://www.ncbi.nlm.nih.gov/genome/proteins/157?project_id=58759) | 0.0683 |
| Bacillus megaterium WSH-002 | Extracellular | 4 | [2049.00](http://www.ncbi.nlm.nih.gov/genome/proteins/945?project_id=74443) | 0.1952 |
| Bacillus thuringiensis Al Hakam | Extracellular | 4 | [4798.00](http://www.ncbi.nlm.nih.gov/genome/proteins/486?project_id=58795) | 0.0834 |
| Bacillus thuringiensis BMB171 | Extracellular | 4 | [5352.00](http://www.ncbi.nlm.nih.gov/genome/proteins/486?project_id=49135) | 0.0747 |
| Bacillus thuringiensis konkukian (serovar konkukian str. 97-27) | Extracellular | 5 | [5197.00](http://www.ncbi.nlm.nih.gov/genome/proteins/486?project_id=58089) | 0.0962 |
| Bacillus thuringiensis serovar chinensis CT-43 | Extracellular | 5 | [6206.00](http://www.ncbi.nlm.nih.gov/genome/proteins/486?project_id=158151) | 0.0806 |
| Bacillus thuringiensis serovar finitimus YBT-020 | Extracellular | 4 | [5782.00](http://www.ncbi.nlm.nih.gov/genome/proteins/486?project_id=158875) | 0.0692 |
| Bacillus weihenstephanensis KBAB4 | Extracellular | 5 | [5653.00](http://www.ncbi.nlm.nih.gov/genome/proteins/1320?project_id=58315) | 0.0884 |
| Brevibacillus brevis NBRC 100599 | Extracellular | 5 | [5947.00](http://www.ncbi.nlm.nih.gov/genome/proteins/1737?project_id=59175) | 0.0841 |
| Candidatus Accumulibacter phosphatis clade IIA str. UW-1 h | Extracellular | 5 | [4562.00](http://www.ncbi.nlm.nih.gov/genome/proteins/1749?project_id=59207) | 0.1096 |
| Candidatus Methylomirabilis oxyfera | Extracellular | 8 | [3072.00](http://www.ncbi.nlm.nih.gov/genome/proteins/12687?project_id=161981) | 0.2604 |
| Candidatus Solibacter usitatus Ellin6076 | Extracellular | 16 | [7826.00](http://www.ncbi.nlm.nih.gov/genome/proteins/1167?project_id=58139) | 0.2044 |
| Cellulophaga algicola DSM 14237 | Extracellular | 15 | [4163.00](http://www.ncbi.nlm.nih.gov/genome/proteins/2470?project_id=62159) | 0.3603 |
| Chitinophaga pinensis DSM 2588 | Extracellular | 6 | [7192.00](http://www.ncbi.nlm.nih.gov/genome/proteins/3398?project_id=59113) | 0.0834 |
| Coraliomargarita akajimensis DSM 45221 | Extracellular | 4 | [3120.00](http://www.ncbi.nlm.nih.gov/genome/proteins/1876?project_id=47079) | 0.1282 |
| Corallococcus coralloides DSM 2259 | Extracellular | 6 | [8033.00](http://www.ncbi.nlm.nih.gov/genome/proteins/12529?project_id=157997) | 0.0747 |
| Corynebacterium glutamicum ATCC 13032 Bielefeld | Extracellular | 4 | [3057.00](http://www.ncbi.nlm.nih.gov/genome/proteins/469?project_id=61611) | 0.1308 |
| Corynebacterium glutamicum ATCC 13032 Kitasato | Extracellular | 4 | [2993.00](http://www.ncbi.nlm.nih.gov/genome/proteins/469?project_id=57905) | 0.1336 |
| Corynebacterium glutamicum R | Extracellular | 4 | [3080.00](http://www.ncbi.nlm.nih.gov/genome/proteins/469?project_id=58897) | 0.1299 |
| Cupriavidus necator N-1 | Extracellular | 5 | [7832.00](http://www.ncbi.nlm.nih.gov/genome/proteins/2490?project_id=68689) | 0.0638 |
| Dechloromonas aromatica RCB | Extracellular | 6 | [4171.00](http://www.ncbi.nlm.nih.gov/genome/proteins/1642?project_id=58025) | 0.1439 |
| Dechlorosoma suillum PS | Extracellular | 4 | [3443.00](http://www.ncbi.nlm.nih.gov/genome/proteins/11148?project_id=81439) | 0.1162 |
| Deferribacter desulfuricans SSM1 | Extracellular | 5 | [2374.00](http://www.ncbi.nlm.nih.gov/genome/proteins/2764?project_id=46653) | 0.2106 |
| Deinococcus gobiensis I-0 | Extracellular | 5 | [4340.00](http://www.ncbi.nlm.nih.gov/genome/proteins/11299?project_id=162509) | 0.1152 |
| Desulfatibacillum alkenivorans AK-01 | Extracellular | 4 | [5252.00](http://www.ncbi.nlm.nih.gov/genome/proteins/1574?project_id=58913) | 0.0762 |
| Desulfococcus oleovorans Hxd3 | Extracellular | 5 | [3265.00](http://www.ncbi.nlm.nih.gov/genome/proteins/1471?project_id=58777) | 0.1531 |
| Desulfomonile tiedjei DSM 6799 | Extracellular | 42 | [5494.00](http://www.ncbi.nlm.nih.gov/genome/proteins/2608?project_id=168320) | 0.7645 |
| Dickeya zeae Ech1591 | Extracellular | 5 | [4163.00](http://www.ncbi.nlm.nih.gov/genome/proteins/1803?project_id=59297) | 0.1201 |
| Dyadobacter fermentans | Extracellular | 6 | [5719.00](http://www.ncbi.nlm.nih.gov/genome/proteins/3389?project_id=59049) | 0.1049 |
| Escherichia coli E24377A | Extracellular | 4 | [4991.00](http://www.ncbi.nlm.nih.gov/genome/proteins/167?project_id=58395) | 0.0801 |
| Escherichia coli O157:H7 str. EC4115 | Extracellular | 4 | [5477.00](http://www.ncbi.nlm.nih.gov/genome/proteins/167?project_id=59091) | 0.0730 |
| Escherichia coli O157:H7 str. TW14359 h | Extracellular | 4 | [5365.00](http://www.ncbi.nlm.nih.gov/genome/proteins/167?project_id=59235) | 0.0746 |
| Flavobacterium johnsoniae UW101 | Extracellular | 6 | [5017.00](http://www.ncbi.nlm.nih.gov/genome/proteins/1391?project_id=58493) | 0.1196 |
| Gemmatimonas aurantiaca T-27 | Extracellular | 8 | [3935.00](http://www.ncbi.nlm.nih.gov/genome/proteins/1632?project_id=58813) | 0.2033 |
| Geobacter metallireducens GS-15 | Extracellular | 5 | [3534.00](http://www.ncbi.nlm.nih.gov/genome/proteins/1038?project_id=57731) | 0.1415 |
| Geobacter sp. M21 | Extracellular | 7 | [4080.00](http://www.ncbi.nlm.nih.gov/genome/proteins/13662?project_id=59037) | 0.1716 |
| Granulicella mallensis MP5ACTX8 | Extracellular | 6 | [4815.00](http://www.ncbi.nlm.nih.gov/genome/proteins/2833?project_id=49957) | 0.1246 |
| Hahella chejuensis KCTC 2396 | Extracellular | 6 | [6773.00](http://www.ncbi.nlm.nih.gov/genome/proteins/1385?project_id=58483) | 0.0886 |
| Haliscomenobacter hydrossis DSM 1100 | Extracellular | 4 | [6752.00](http://www.ncbi.nlm.nih.gov/genome/proteins/2873?project_id=66777) | 0.0592 |
| Lysinibacillus sphaericus C3-41 | Extracellular | 4 | [4771.00](http://www.ncbi.nlm.nih.gov/genome/proteins/1582?project_id=58945) | 0.0838 |
| Magnetococcus marinus MC-1 | Extracellular | 4 | [3716.00](http://www.ncbi.nlm.nih.gov/genome/proteins/1064?project_id=57833) | 0.1076 |
| Myxococcus fulvus HW-1 | Extracellular | 4 | [7284.00](http://www.ncbi.nlm.nih.gov/genome/proteins/1701?project_id=68443) | 0.0549 |
| Myxococcus xanthus DK 1622 | Extracellular | 7 | [7316.00](http://www.ncbi.nlm.nih.gov/genome/proteins/1120?project_id=58003) | 0.0957 |
| Niastella koreensis GR20-10 | Extracellular | 4 | [7174.00](http://www.ncbi.nlm.nih.gov/genome/proteins/2567?project_id=83125) | 0.0558 |
| Paenibacillus mucilaginosus 3016 | Extracellular | 4 | [7057.00](http://www.ncbi.nlm.nih.gov/genome/proteins/3587?project_id=89377) | 0.0567 |
| Paenibacillus sp. Y412MC10 | Extracellular | 4 | [6238.00](http://www.ncbi.nlm.nih.gov/genome/proteins/13690?project_id=41127) | 0.0641 |
| Pedobacter saltans DSM 12145 | Extracellular | 6 | [3792.00](http://www.ncbi.nlm.nih.gov/genome/proteins/2918?project_id=61349) | 0.1582 |
| Persephonella marina EX-H1 | Extracellular | 4 | [2051.00](http://www.ncbi.nlm.nih.gov/genome/proteins/1151?project_id=58119) | 0.1950 |
| Phycisphaera mikurensis NBRC 102666 | Extracellular | 4 | [3282.00](http://www.ncbi.nlm.nih.gov/genome/proteins/12626?project_id=157331) | 0.1219 |
| Planctomyces brasiliensis DSM 5305 | Extracellular | 4 | [4750.00](http://www.ncbi.nlm.nih.gov/genome/proteins/2824?project_id=60583) | 0.0842 |
| Polaromonas naphthalenivorans CJ2 | Extracellular | 4 | [4929.00](http://www.ncbi.nlm.nih.gov/genome/proteins/1236?project_id=58273) | 0.0812 |
| Pseudomonas protegens Pf-5 | Extracellular | 5 | [6108.00](http://www.ncbi.nlm.nih.gov/genome/proteins/15290?project_id=57937) | 0.0819 |
| Pseudovibrio sp. FO-BEG1 | Extracellular | 4 | [5468.00](http://www.ncbi.nlm.nih.gov/genome/proteins/13683?project_id=82373) | 0.0732 |
| Pseudoxanthomonas spadix BD-a59 | Extracellular | 4 | [3149.00](http://www.ncbi.nlm.nih.gov/genome/proteins/11569?project_id=75113) | 0.1270 |
| Ralstonia solanacearum GMI1000 | Extracellular | 5 | [5113.00](http://www.ncbi.nlm.nih.gov/genome/proteins/490?project_id=57593) | 0.0978 |
| Ralstonia solanacearum Po82 | Extracellular | 5 | [5016.00](http://www.ncbi.nlm.nih.gov/genome/proteins/490?project_id=162133) | 0.0997 |
| Ralstonia solanacearum PSI07 | Extracellular | 8 | [4978.00](http://www.ncbi.nlm.nih.gov/genome/proteins/490?project_id=50539) | 0.1607 |
| Rhodopirellula baltica SH 1 | Extracellular | 9 | [7325.00](http://www.ncbi.nlm.nih.gov/genome/proteins/1094?project_id=61589) | 0.1229 |
| Robiginitalea biformata HTCC2501 | Extracellular | 5 | [3209.00](http://www.ncbi.nlm.nih.gov/genome/proteins/1668?project_id=58285) | 0.1558 |
| Shewanella woodyi ATCC 51908 | Extracellular | 5 | [4880.00](http://www.ncbi.nlm.nih.gov/genome/proteins/1457?project_id=58721) | 0.1025 |
| Sideroxydans lithotrophicus ES-1 | Extracellular | 6 | [2980.00](http://www.ncbi.nlm.nih.gov/genome/proteins/1862?project_id=46801) | 0.2013 |
| Slackia heliotrinireducens DSM 20476 | Extracellular | 4 | [2766.00](http://www.ncbi.nlm.nih.gov/genome/proteins/3390?project_id=59051) | 0.1446 |
| Solitalea canadensis DSM 3403 | Extracellular | 6 | [4310.00](http://www.ncbi.nlm.nih.gov/genome/proteins/10812?project_id=81783) | 0.1392 |
| Spirochaeta africana | Extracellular | 4 | [2782.00](http://www.ncbi.nlm.nih.gov/genome/proteins/11382?project_id=81779) | 0.1438 |
| Stenotrophomonas maltophilia JV3 | Extracellular | 4 | [4063.00](http://www.ncbi.nlm.nih.gov/genome/proteins/880?project_id=72473) | 0.0984 |
| Stenotrophomonas maltophilia K279a h | Extracellular | 7 | [4386.00](http://www.ncbi.nlm.nih.gov/genome/proteins/880?project_id=61647) | 0.1596 |
| Stigmatella aurantiaca DW4/3-1 | Extracellular | 4 | [8543.00](http://www.ncbi.nlm.nih.gov/genome/proteins/1355?project_id=54333) | 0.0468 |
| Streptomyces bingchenggensis BCW-1 | Extracellular | 4 | [10022.00](http://www.ncbi.nlm.nih.gov/genome/proteins/2825?project_id=82931) | 0.0399 |
| Streptomyces flavogriseus ATCC 33331 | Extracellular | 6 | [6572.00](http://www.ncbi.nlm.nih.gov/genome/proteins/1902?project_id=40839) | 0.0913 |
| Streptomyces scabiei 87.22 | Extracellular | 5 | [8746.00](http://www.ncbi.nlm.nih.gov/genome/proteins/1178?project_id=46531) | 0.0572 |
| Streptosporangium roseum DSM 43021 | Extracellular | 5 | [8975.00](http://www.ncbi.nlm.nih.gov/genome/proteins/3368?project_id=42521) | 0.0557 |
| Sulfuricurvum kujiense DSM 16994 | Extracellular | 5 | [2798.00](http://www.ncbi.nlm.nih.gov/genome/proteins/2682?project_id=60789) | 0.1787 |
| Thauera sp. MZ1T | Extracellular | 6 | [3978.00](http://www.ncbi.nlm.nih.gov/genome/proteins/13688?project_id=58987) | 0.1508 |
| Trichodesmium erythraeum IMS101 | Extracellular | 4 | [4451.00](http://www.ncbi.nlm.nih.gov/genome/proteins/1080?project_id=57925) | 0.0899 |
| Variovorax paradoxus EPS | Extracellular | 4 | [5952.00](http://www.ncbi.nlm.nih.gov/genome/proteins/1766?project_id=62107) | 0.0672 |
| Xanthomonas axonopodis pv. citri 306 | Extracellular | 4 | [4427.00](http://www.ncbi.nlm.nih.gov/genome/proteins/527?project_id=57889) | 0.0904 |
| Xanthomonas axonopodis pv. citrumelo F1 | Extracellular | 6 | [4181.00](http://www.ncbi.nlm.nih.gov/genome/proteins/10698?project_id=73179) | 0.1435 |
| Xanthomonas campestris 8004 | Extracellular | 9 | [4271.00](http://www.ncbi.nlm.nih.gov/genome/proteins/151?project_id=57595) | 0.2107 |
| Xanthomonas campestris pv. campestris ATCC33913 | Extracellular | 7 | [4179.00](http://www.ncbi.nlm.nih.gov/genome/proteins/151?project_id=57887) | 0.1675 |
| Xanthomonas campestris pv. campestris str. B100 | Extracellular | 7 | [4466.00](http://www.ncbi.nlm.nih.gov/genome/proteins/151?project_id=61643) | 0.1567 |
| Xanthomonas campestris pv. raphani 756C | Extracellular | 9 | [4516.00](http://www.ncbi.nlm.nih.gov/genome/proteins/151?project_id=159539) | 0.1993 |
| Xanthomonas campestris pv. vesicatoria str. 85-10 | Extracellular | 4 | [4726.00](http://www.ncbi.nlm.nih.gov/genome/proteins/2508?project_id=58321) | 0.0846 |
| Zobellia galactanivorans | Extracellular | 4 | [4732.00](http://www.ncbi.nlm.nih.gov/genome/proteins/1396?project_id=70621) | 0.0845 |
| Acaryochloris marina MBIC11017 | Facultative | 12 | [8383.00](http://www.ncbi.nlm.nih.gov/genome/proteins/1179?project_id=58167) | 0.1431 |
| Arcobacter butzleri ED-1 | Facultative | 4 | [2158.00](http://www.ncbi.nlm.nih.gov/genome/proteins/1690?project_id=158699) | 0.1854 |
| Arcobacter butzleri RM4018 | Facultative | 4 | [2259.00](http://www.ncbi.nlm.nih.gov/genome/proteins/1690?project_id=58557) | 0.1771 |
| Bacteriovorax marinus SJ | Facultative | 4 | [3231.00](http://www.ncbi.nlm.nih.gov/genome/proteins/1160?project_id=82341) | 0.1238 |
| Bdellovibrio bacteriovorus HD100 | Facultative | 4 | [3586.00](http://www.ncbi.nlm.nih.gov/genome/proteins/1643?project_id=61595) | 0.1115 |
| Brachyspira hyodysenteriae WA1 | Facultative | 60 | [2642.00](http://www.ncbi.nlm.nih.gov/genome/proteins/1801?project_id=59291) | 2.2710 |
| Brachyspira intermedia PWS/A | Facultative | 57 | [2872.00](http://www.ncbi.nlm.nih.gov/genome/proteins/6920?project_id=158369) | 1.9847 |
| Brachyspira murdochii DSM 12563 | Facultative | 48 | [2809.00](http://www.ncbi.nlm.nih.gov/genome/proteins/3377?project_id=48819) | 1.7088 |
| Brachyspira pilosicoli 95/1000 | Facultative | 32 | [2299.00](http://www.ncbi.nlm.nih.gov/genome/proteins/2885?project_id=50609) | 1.3919 |
| Burkholderia cenocepacia HI2424 | Facultative | 4 | [6919.00](http://www.ncbi.nlm.nih.gov/genome/proteins/475?project_id=58369) | 0.0578 |
| Burkholderia cenocepacia J2315 | Facultative | 6 | [7116.00](http://www.ncbi.nlm.nih.gov/genome/proteins/475?project_id=57953) | 0.0843 |
| Burkholderia cenocepacia MC0-3 | Facultative | 5 | [7008.00](http://www.ncbi.nlm.nih.gov/genome/proteins/475?project_id=58769) | 0.0713 |
| Burkholderia glumae BGR1 | Facultative | 5 | [5773.00](http://www.ncbi.nlm.nih.gov/genome/proteins/946?project_id=59397) | 0.0866 |
| Burkholderia rhizoxinica HKI 454 | Facultative | 4 | [3870.00](http://www.ncbi.nlm.nih.gov/genome/proteins/3327?project_id=60487) | 0.1034 |
| Burkholderia sp. 383 | Facultative | 6 | [7716.00](http://www.ncbi.nlm.nih.gov/genome/proteins/1135?project_id=58073) | 0.0778 |
| Burkholderia sp. CCGE1002 | Facultative | 5 | [6889.00](http://www.ncbi.nlm.nih.gov/genome/proteins/1135?project_id=42523) | 0.0726 |
| Burkholderia sp. KJ006 | Facultative | 5 | [6024.00](http://www.ncbi.nlm.nih.gov/genome/proteins/1135?project_id=165871) | 0.0830 |
| Burkholderia vietnamiensis G4 | Facultative | 37 | [7617.00](http://www.ncbi.nlm.nih.gov/genome/proteins/1136?project_id=58075) | 0.4858 |
| Burkholderia xenovorans LB400 | Facultative | 6 | [8702.00](http://www.ncbi.nlm.nih.gov/genome/proteins/1063?project_id=57823) | 0.0689 |
| Elusimicrobium minutum | Facultative | 5 | [1529.00](http://www.ncbi.nlm.nih.gov/genome/proteins/1584?project_id=58949) | 0.3270 |
| Francisella cf. novicida 3523 | Facultative | 5 | [1854.00](http://www.ncbi.nlm.nih.gov/genome/proteins/906?project_id=162107) | 0.2697 |
| Helicobacter hepaticus ATCC 51449 | Facultative | 13 | [1876.00](http://www.ncbi.nlm.nih.gov/genome/proteins/1102?project_id=57737) | 0.6930 |
| Legionella longbeachae NSW150 | Facultative | 26 | [3470.00](http://www.ncbi.nlm.nih.gov/genome/proteins/1393?project_id=46099) | 0.7493 |
| Legionella pneumophila 2300/99 Alcoy h | Facultative | 17 | [3190.00](http://www.ncbi.nlm.nih.gov/genome/proteins/416?project_id=48801) | 0.5329 |
| Legionella pneumophila Lens | Facultative | 19 | [2934.00](http://www.ncbi.nlm.nih.gov/genome/proteins/416?project_id=58209) | 0.6476 |
| Legionella pneumophila Paris | Facultative | 21 | [3166.00](http://www.ncbi.nlm.nih.gov/genome/proteins/416?project_id=58211) | 0.6633 |
| Legionella pneumophila Philadelphia 1 | Facultative | 15 | [2943.00](http://www.ncbi.nlm.nih.gov/genome/proteins/416?project_id=57609) | 0.5097 |
| Legionella pneumophila str. Corby | Facultative | 17 | [3204.00](http://www.ncbi.nlm.nih.gov/genome/proteins/416?project_id=58733) | 0.5306 |
| Legionella pneumophila subsp. pneumophila ATCC 43290 | Facultative | 15 | [2926.00](http://www.ncbi.nlm.nih.gov/genome/proteins/416?project_id=86885) | 0.5126 |
| Leptospira biflexa serovar Patoc strain 'Patoc 1 (Ames)' | Facultative | 15 | [3600.00](http://www.ncbi.nlm.nih.gov/genome/proteins/750?project_id=58511) | 0.4167 |
| Leptospira biflexa serovar Patoc strain 'Patoc 1 (Paris)' | Facultative | 15 | [3726.00](http://www.ncbi.nlm.nih.gov/genome/proteins/750?project_id=58993) | 0.4026 |
| Leptospira borgpetersenii sv Hardjo-bovis JB197 | Facultative | 8 | [2880.00](http://www.ncbi.nlm.nih.gov/genome/proteins/519?project_id=58509) | 0.2778 |
| Leptospira borgpetersenii sv Hardjo-bovis L550 | Facultative | 5 | [2945.00](http://www.ncbi.nlm.nih.gov/genome/proteins/519?project_id=58507) | 0.1698 |
| Leptospira interrogans sv Copenhageni Fiocruz L1-130 | Facultative | 13 | [3667.00](http://www.ncbi.nlm.nih.gov/genome/proteins/179?project_id=58065) | 0.3545 |
| Leptospira interrogans sv Lai 56601 | Facultative | 15 | [3702.00](http://www.ncbi.nlm.nih.gov/genome/proteins/179?project_id=57881) | 0.4052 |
| Leptospira interrogans serovar Lai str. IPAV | Facultative | 15 | 3711 | 0.4042 |
| Shigella flexneri 2002017 | Facultative | 4 | [4703.00](http://www.ncbi.nlm.nih.gov/genome/proteins/182?project_id=159233) | 0.0851 |
| Shigella sonnei 53G | Facultative | 5 | [5412.00](http://www.ncbi.nlm.nih.gov/genome/proteins/417?project_id=84383) | 0.0924 |
| Sphingobacterium sp. 21 h | Facultative | 6 | [5169.00](http://www.ncbi.nlm.nih.gov/genome/proteins/13771?project_id=64755) | 0.1161 |
| Treponema denticola ATCC 35405 | Facultative | 6 | [2767.00](http://www.ncbi.nlm.nih.gov/genome/proteins/1001?project_id=57583) | 0.2168 |
| Treponema azotonutricium ZAS-9 | Facultative | 5 | [3474.00](http://www.ncbi.nlm.nih.gov/genome/proteins/1763?project_id=67365) | 0.1439 |
| Treponema succinifaciens DSM 2489 | Facultative | 5 | [2608.00](http://www.ncbi.nlm.nih.gov/genome/proteins/3024?project_id=65781) | 0.1917 |
| Turneriella parva DSM 21527 | Facultative | 21 | [4139.00](http://www.ncbi.nlm.nih.gov/genome/proteins/11358?project_id=168321) | 0.5074 |
| Anaplasma phagocytophilum HZ | Obligate | 4 | [1264.00](http://www.ncbi.nlm.nih.gov/genome/proteins/1083?project_id=57951) | 0.3165 |
| Candidatus Amoebophilus asiaticus 5a2 | Obligate | 46 | [1334.00](http://www.ncbi.nlm.nih.gov/genome/proteins/1590?project_id=58963) | 3.4483 |
| Candidatus Midichloria mitochondrii IricVA | Obligate | 8 | [1211.00](http://www.ncbi.nlm.nih.gov/genome/proteins/2543?project_id=68687) | 0.6606 |
| Candidatus Protochlamydia amoebophila UWE25 | Obligate | 6 | [2031.00](http://www.ncbi.nlm.nih.gov/genome/proteins/1137?project_id=58079) | 0.2954 |
| Candidatus Rickettsia amblyommii str. GAT-30V | Obligate | 9 | [1390.00](http://www.ncbi.nlm.nih.gov/genome/proteins/3680?project_id=156845) | 0.6475 |
| Cardinium hertigii cEper1 | Obligate | *19* | 879.00 | 2.1615 |
| Coxiella burnetii CbuG_Q212 | Obligate | 9 | [1866.00](http://www.ncbi.nlm.nih.gov/genome/proteins/543?project_id=58893) | 0.4823 |
| Coxiella burnetii CbuK_Q154 | Obligate | 10 | [1942.00](http://www.ncbi.nlm.nih.gov/genome/proteins/543?project_id=58895) | 0.5149 |
| Coxiella burnetii Dugway 7E9-12 (5J108-111) | Obligate | 16 | [2045.00](http://www.ncbi.nlm.nih.gov/genome/proteins/543?project_id=58629) | 0.7824 |
| Coxiella burnetii RSA 331 | Obligate | 13 | [1975.00](http://www.ncbi.nlm.nih.gov/genome/proteins/543?project_id=58637) | 0.6582 |
| Coxiella burnetii RSA 493 | Obligate | 8 | [1847.00](http://www.ncbi.nlm.nih.gov/genome/proteins/543?project_id=57631) | 0.4331 |
| Ehrlichia canis Jake | Obligate | 5 | [925.00](http://www.ncbi.nlm.nih.gov/genome/proteins/1134?project_id=58071) | 0.5405 |
| Ehrlichia chaffeensis Arkansas | Obligate | 5 | [1105.00](http://www.ncbi.nlm.nih.gov/genome/proteins/482?project_id=57933) | 0.4525 |
| Ehrlichia ruminantium Gardel | Obligate | 5 | [950.00](http://www.ncbi.nlm.nih.gov/genome/proteins/436?project_id=58245) | 0.5263 |
| Ehrlichia ruminantium Welgevonden France | Obligate | 4 | [888.00](http://www.ncbi.nlm.nih.gov/genome/proteins/436?project_id=58013) | 0.4505 |
| Ehrlichia ruminantium Welgevonden South Africa | Obligate | 4 | [958.00](http://www.ncbi.nlm.nih.gov/genome/proteins/436?project_id=58243) | 0.4175 |
| Neorickettsia risticii str. Illinois | Obligate | 4 | [892.00](http://www.ncbi.nlm.nih.gov/genome/proteins/1513?project_id=58889) | 0.4484 |
| Orientia tsutsugamushi str. Boryong | Obligate | 37 | [1182.00](http://www.ncbi.nlm.nih.gov/genome/proteins/710?project_id=61621) | 3.1303 |
| Orientia tsutsugamushi str. Ikeda | Obligate | 47 | [1967.00](http://www.ncbi.nlm.nih.gov/genome/proteins/710?project_id=58869) | 2.3894 |
| Parachlamydia acanthamoebae UV-7 | Obligate | 18 | [2789.00](http://www.ncbi.nlm.nih.gov/genome/proteins/2157?project_id=68335) | 0.6454 |
| Rickettsia africae ESF-5 | Obligate | 4 | [1041.00](http://www.ncbi.nlm.nih.gov/genome/proteins/1477?project_id=58799) | 0.3842 |
| Rickettsia akari str. Hartford | Obligate | 7 | [1258.00](http://www.ncbi.nlm.nih.gov/genome/proteins/1177?project_id=58161) | 0.5564 |
| Rickettsia australis str. Cutlack | Obligate | 7 | [1261.00](http://www.ncbi.nlm.nih.gov/genome/proteins/11869?project_id=158039) | 0.5551 |
| Rickettsia bellii OSU 85-389 | Obligate | 28 | [1475.00](http://www.ncbi.nlm.nih.gov/genome/proteins/661?project_id=58681) | 1.8983 |
| Rickettsia bellii RML369-C | Obligate | 27 | [1429.00](http://www.ncbi.nlm.nih.gov/genome/proteins/661?project_id=58405) | 1.8894 |
| Rickettsia canadensis str. CA410 | Obligate | 4 | [1016.00](http://www.ncbi.nlm.nih.gov/genome/proteins/1176?project_id=88063) | 0.3937 |
| Rickettsia canadensis str. McKiel | Obligate | 4 | [1090.00](http://www.ncbi.nlm.nih.gov/genome/proteins/1176?project_id=58159) | 0.3670 |
| Rickettsia conorii Malish 7 | Obligate | 6 | [1374.00](http://www.ncbi.nlm.nih.gov/genome/proteins/1011?project_id=57633) | 0.4367 |
| Rickettsia felis URRWXCal2 | Obligate | 24 | [1512.00](http://www.ncbi.nlm.nih.gov/genome/proteins/1330?project_id=58331) | 1.5873 |
| Rickettsia heilongjiangensis 054 | Obligate | 11 | [1297.00](http://www.ncbi.nlm.nih.gov/genome/proteins/6628?project_id=70839) | 0.8481 |
| Rickettsia japonica YH | Obligate | 8 | [971.00](http://www.ncbi.nlm.nih.gov/genome/proteins/11157?project_id=73963) | 0.8239 |
| Rickettsia massiliae MTU5 | Obligate | 5 | [980.00](http://www.ncbi.nlm.nih.gov/genome/proteins/1478?project_id=58801) | 0.5102 |
| Rickettsia massiliae str. AZT80 | Obligate | 6 | [1207.00](http://www.ncbi.nlm.nih.gov/genome/proteins/1478?project_id=86751) | 0.4971 |
| Rickettsia montanensis str. OSU 85-930 | Obligate | 6 | [1217.00](http://www.ncbi.nlm.nih.gov/genome/proteins/11871?project_id=158043) | 0.4930 |
| Rickettsia parkeri str. Portsmouth | Obligate | 9 | [1318.00](http://www.ncbi.nlm.nih.gov/genome/proteins/11868?project_id=158045) | 0.6829 |
| Rickettsia philipii str. 364D | Obligate | 8 | [1344.00](http://www.ncbi.nlm.nih.gov/genome/proteins/11867?project_id=89383) | 0.5952 |
| Rickettsia rhipicephali str. 3-7-female6-CWPP | Obligate | 7 | [1266.00](http://www.ncbi.nlm.nih.gov/genome/proteins/11870?project_id=156977) | 0.5529 |
| Rickettsia rickettsii str. 'Sheila Smith' | Obligate | 6 | [1343.00](http://www.ncbi.nlm.nih.gov/genome/proteins/674?project_id=58027) | 0.4468 |
| Rickettsia rickettsii str. Arizona | Obligate | 6 | [1343.00](http://www.ncbi.nlm.nih.gov/genome/proteins/674?project_id=86655) | 0.4468 |
| Rickettsia rickettsii str. Brazil | Obligate | 6 | [1332.00](http://www.ncbi.nlm.nih.gov/genome/proteins/674?project_id=88069) | 0.4505 |
| Rickettsia rickettsii str. Colombia | Obligate | 6 | [1350.00](http://www.ncbi.nlm.nih.gov/genome/proteins/674?project_id=86653) | 0.4444 |
| Rickettsia rickettsii str. Hauke | Obligate | 6 | [1340.00](http://www.ncbi.nlm.nih.gov/genome/proteins/674?project_id=86659) | 0.4478 |
| Rickettsia rickettsii str. Hino | Obligate | 6 | [1335.00](http://www.ncbi.nlm.nih.gov/genome/proteins/674?project_id=86657) | 0.4494 |
| Rickettsia rickettsii str. Hlp#2 | Obligate | 6 | [1308.00](http://www.ncbi.nlm.nih.gov/genome/proteins/674?project_id=88067) | 0.4587 |
| Rickettsia rickettsii str. Iowa | Obligate | 6 | [1384.00](http://www.ncbi.nlm.nih.gov/genome/proteins/674?project_id=58961) | 0.4335 |
| Rickettsia slovaca 13-B | Obligate | 5 | [1112.00](http://www.ncbi.nlm.nih.gov/genome/proteins/11089?project_id=82369) | 0.4496 |
| Rickettsia slovaca str. D-CWPP | Obligate | 7 | [1347.00](http://www.ncbi.nlm.nih.gov/genome/proteins/11089?project_id=158159) | 0.5197 |
| Simkania negevensis Z | Obligate | 13 | [2518.00](http://www.ncbi.nlm.nih.gov/genome/proteins/1152?project_id=68451) | 0.5163 |
| Waddlia chondrophila WSU 86-1044 | Obligate | 8 | [1956.00](http://www.ncbi.nlm.nih.gov/genome/proteins/2757?project_id=49531) | 0.4090 |
| Wolbachia endosymbiont of Culex quinquefasciatus Pel | Obligate | 58 | [1275.00](http://www.ncbi.nlm.nih.gov/genome/proteins/1164?project_id=61645) | 4.5490 |
| Wolbachia pipientis wBm | Obligate | 7 | [1058.00](http://www.ncbi.nlm.nih.gov/genome/proteins/11990?project_id=81759) | 0.6616 |
| Wolbachia pipientis wMel | Obligate | 24 | [1195.00](http://www.ncbi.nlm.nih.gov/genome/proteins/1065?project_id=57851) | 2.0084 |
| Wolbachia sp. wRi | Obligate | 31 | [1150.00](http://www.ncbi.nlm.nih.gov/genome/proteins/13706?project_id=59371) | 2.6957 |
