# Supplementary material for: Ankyrin domains across the Tree of Life
Source: PeerJ. 2014 Feb 6;2:e264. doi: 10.7717/peerj.264 (PMC3932732; doi:10.7717/peerj.264)
Supplement: Supplemental Information 6 [file peerj-02-264-s006.pdf]

Lifestyle	Genome	Class/Order	Ankryin proteins	Total Gene #	% Genome
Extracellular	*Xanthomonas campestris 8004*	*Gammaproteobacteria; Xanthomonadales*	9	4,227	0.213
	Ralstonia solanacearum PSI07	*Betaproteobacteria; Burkholderiales*	8	7,457	0.107
	Geobacter sp. M21	*Deltaproteobacteria; Desulfuromonadales*	7	4,204	0.167
	Myxococcus xanthus DK 1622	*Deltaproteobacteria; Myxococcales*	7	6,781	0.103
	Hahella chejuensis KCTC 2396	*Gammaproteobacteria; Oceanospirillales*	6	6,273	0.096
	Bacillus cereus E33L	*Bacilli; Bacillales*	5	5,796	0.086
	Acinetobacter baumannii 1656-2	*Gammaproteobacteria; Pseudomonadales*	4	3,913	0.102
	Actinomyces graevenitzii C83	*Actinobacteria; Actinobacteridae*	4	1,910	0.209
	Agrobacterium radiobacter K84	*Alphaproteobacteria; Rhizobiales*	4	6,820	0.059
	Anabaena variabilis ATCC 29413	*Nostocales; Nostocaceae*	4	5697	0.070
	Chitinophaga pinensis DSM 2588	*Bacteroidetes; Sphingobacteriia*	6	7,399	0.081
	Coraliomargarita akajimensis DSM 45221	*Verrucomicrobia; Opitutae*	4	3,192	0.125
	Deferribacter desulfuricans SSM1	*Deferribacteres; Deferribacterales*	5	2,442	0.205
	Deinococcus gobiensis I-0	*Deinococci; Deinococcales*	5	4,465	0.112
	Gemmatimonas aurantiaca T-27	*Gemmatimonadetes; Gemmatimonadales*	8	3,989	0.201
	Persephonella marina EX-H1	*Aquificae; Aquificales;*	4	2,095	0.191
	Phycisphaera mikurensis NBRC 102666	*Phycisphaerae; Phycisphaerales*	4	3,334	0.120
	Planctomyces brasiliensis DSM 5305	*Planctomycetia; Planctomycetales*	4	4,865	0.082
	Spirochaeta africana	*Spirochaetia; Spirochaetales*	4	2,873	0.139
	Sulfuricurvum kujiense DSM 16994	*Epsilonproteobacteria; Campylobacterales*	5	2,879	0.174
	Trichodesmium erythraeum IMS101	*Oscillatoriales; Trichodesmium*	4	5076	0.079
Facultative	*Burkholderia vietnamiensis G4*	*Betaproteobacteria; Burkholderiales*	37	7,775	0.476
	Legionella pneumophila Philadelphia 1	*Gammaproteobacteria; Legionellales*	15	2,942	0.510
	Leptospira biflexa serovar Patoc strain 'Patoc 1 (Paris)'	*Spirochaetia; Spirochaetales*	15	3,775	0.397
	Helicobacter hepaticus ATCC 51449	*Epsilonproteobacteria; Campylobacterales*	13	1,916	0.678
	Francisella cf. novicida 3523	*Gammaproteobacteria; Thiotrichales*	5	1,898	0.263
	Bacteriovorax marinus SJ	*Deltaproteobacteria; Bdellovibrionales*	4	3,292	0.122
	Elusimicrobium minutum	*Elusimicrobia; Elusimicrobiales*	5	1,599	0.313
	Francisella cf. novicida 3523	*Gammaproteobacteria; Thiotrichales*	5	1898	0.263
	Shigella flexneri 2002017	*Gammaproteobacteria; Enterobacteriales*	4	5,058	0.079
Obligate	*Wolbachia pipientis wMel*	*Alphaproteobacteria; Rickettsiales*	24	1,308	1.835
	Cardinium hertigii cEper1	*Bacteroidetes; Bacteroidales*	19	879	2.162
	Coxiella burnetii Dugway 7E9-12	*Gammaproteobacteria; Legionellales*	16	2,296	0.697
	Candidatus Protochlamydia amoebophila UWE25	*Chlamydiae; Chlamydiia*	6	2,031	0.295
	Treponema pallidum pallidum Nichols	*Spirochaetia; Spirochaetales*	2	1,095	0.183
	Acaryochloris marina MBIC11017	*Chroococcales; Acaryochloris*	12	8,571	0.140
