# Supplementary material for: Ankyrin domains across the Tree of Life
Source: PeerJ. 2014 Feb 6;2:e264. doi: 10.7717/peerj.264 (PMC3932732; doi:10.7717/peerj.264)
Supplement: Supplemental Information 7 [file peerj-02-264-s007.pdf]

Obligate Intracellular Bacteria	Transmission	# Ankryin proteins	Total Protein	% Proteins with Ank
Anaplasma marginale str. St. Maries	H	3	948	0.3164557
Anaplasma phagocytophilum HZ	H	4	1264	0.3164557
Candidatus Protochlamydia amoebophila UWE25	H	6	2031	0.29542097
Candidatus Ruthia magnifica str. Cm (Calyptogena magnifica)	H	0		0
Chlamydophila pneumoniae AR39	H	0		0
Chlamydophila pneumoniae J138	H	0		0
Chlamydophila pneumoniae TW-183	H	0		0
Chlamydia trachomatis A/HAR-13	H	0		0
Chlamydophila caviae GPIC	H	0		0
Coxiella burnetii CbuG_Q212	H	9	1866	0.48231511
Coxiella burnetii CbuK_Q154	H	10	1942	0.51493306
Coxiella burnetii Dugway 5J108-111	H	16	2045	0.78239609
Ehrlichia canis str. Jake	H	5	925	0.54054054
Ehrlichia chaffeensis str. Arkansas	H	5	1105	0.45248869
Ehrlichia ruminantium str. Gardel	H	5	950	0.52631579
Ehrlichia ruminantium str. Welgevonden	H	4	958	0.41753653
Ehrlichia ruminantium str. Welgevonden	H	4	888	0.45045045
Lawsonia intracellularis PHE/MN1-00	H	0		0
Mycobacterium leprae TN	H	0		0
Mycoplasma capricolum subsp. capricolum ATCC 27343	H	0		0
Mycoplasma mycoides subsp. mycoides SC str. PG1	H	0		0
Neorickettsia sennetsu str. Miyayama	H	3	932	0.32188841
Onion yellows phytoplasma OY-M	H	0		0
Rickettsia bellii RML369-C	H	27	1429	1.88943317
Rickettsia conorii str. Malish 7	H	6	1374	0.43668122
Rickettsia felis URRWXCal2	H	24	1512	1.58730159
Rickettsia prowazekii str. Madrid E	H	3	835	0.35928144
Rickettsia typhi str. Wilmington	H	2	837	0.23894863
Tropheryma whipplei str. Twist	H	0		0
Tropheryma whipplei TW08/27	H	0		0
Wolbachia endosymbiont of Drosophila melanogaster	H	24	1195	2.0083682
Baumannia cicadellinicola str. Hc (Homalodisca coagulata)	V	0		0
Buchnera aphidicola str. Bp (Baizongia pistaciae)	V	0		0
Buchnera aphidicola str. Sg (Schizaphis graminum)	V	0		0
Buchnera aphidicola str. APS (Acyrthosiphon pisum)	V	0		0
Candidatus Blochmannia floridanus	V	0		0
Candidatus Blochmannia pennsylvanicus str. BPEN	V	0		0
Wigglesworthia glossinidia endosymbiont of Glossina brevipalpis	V	0		0
Wolbachia endosymbiont strain TRS of Brugia malayi	V	7	805	0.86956522
