# Supplementary material for: Ankyrin domains across the Tree of Life
Source: PeerJ. 2014 Feb 6;2:e264. doi: 10.7717/peerj.264 (PMC3932732; doi:10.7717/peerj.264)
Supplement: Supplemental Information 8 [file peerj-02-264-s008.pdf]

Lifestyle	Genome	Class/Order	Ankryin proteins	Total Gene Number	% Genome
Extracellular	*Xanthomonas campestris 8004*	*Gammaproteobacteria; Xanthomonadales*	9	4,227	0.213
	Ralstonia solanacearum PSI07	*Betaproteobacteria; Burkholderiales*	8	7,457	0.107
	Geobacter sp. M21	*Deltaproteobacteria; Desulfuromonadales*	7	4,204	0.167
	Myxococcus xanthus DK 1622	*Deltaproteobacteria; Myxococcales*	7	6,781	0.103
	Hahella chejuensis KCTC 2396	*Gammaproteobacteria; Oceanospirillales*	6	6,273	0.096
	Bacillus cereus E33L	*Bacilli; Bacillales*	5	5,796	0.086
Facultative	*Burkholderia vietnamiensis G4*	*Betaproteobacteria; Burkholderiales*	37	7,775	0.476
	Legionella pneumophila Philadelphia 1	*Gammaproteobacteria; Legionellales*	15	2,942	0.510
	Leptospira biflexa serovar Patoc strain 'Patoc 1 (Paris)'	*Spirochaetia; Spirochaetales*	15	3,775	0.397
	Helicobacter hepaticus ATCC 51449	*Epsilonproteobacteria; Campylobacterales*	13	1,916	0.678
	Francisella cf. novicida 3523	*Gammaproteobacteria; Thiotrichales*	5	1,898	0.263
Obligate	*Wolbachia pipientis wMel*	*Alphaproteobacteria; Rickettsiales*	24	1,308	1.835
	Cardinium hertigii cEper1	*Bacteroidetes; Bacteroidales*	19	879	2.162
	Coxiella burnetii Dugway 7E9-12	*Gammaproteobacteria; Legionellales*	16	2,296	0.697
	Candidatus Protochlamydia amoebophila UWE25	*Chlamydiae; Chlamydiia*	6	2,031	0.295
	Treponema pallidum pallidum Nichols	*Spirochaetia; Spirochaetales*	2	1,095	0.183
