# Supplementary material for: Ankyrin domains across the Tree of Life
Source: PeerJ. 2014 Feb 6;2:e264. doi: 10.7717/peerj.264 (PMC3932732; doi:10.7717/peerj.264)
Supplement: Supplemental Information 10 [file peerj-02-264-s010.pdf]

Archaea	# Ankryin proteins	Total Protein #	% Proteins
Acidianus hospitalis W1	1	2329	0.043
Halogeometricum borinquense DSM 11551	4	3880	0.103
Metallosphaera cuprina Ar-4	1	2029	0.049
Methanocella paludicola SANAE	2	3004	0.067
Methanococcus aeolicus Nankai-3	1	1490	0.067
Methanosarcina barkeri str. Fusaro	1	3624	0.028
Methanosarcina mazei Go1	1	3368	0.030
Methanothermococcus okinawensis IH1	1	1595	0.063
Pyrobaculum aerophilum str. IM2	1	2604	0.038
Pyrobaculum arsenaticum DSM 13514	1	2299	0.043
Pyrobaculum oguniense TE7	6	2835	0.212
Sulfolobus islandicus Y.N.15.51	2	2900	0.069
Thermofilum pendens Hrk 5	1	1878	0.053
Thermoplasma acidophilum DSM 1728	1	1484	0.067
Thermoplasma volcanium GSS1	1	1501	0.067
