# Supplementary material for: Ankyrin domains across the Tree of Life
Source: PeerJ. 2014 Feb 6;2:e264. doi: 10.7717/peerj.264 (PMC3932732; doi:10.7717/peerj.264)
Supplement: Supplemental Information 11 — TM, transmemebrane domain. The number in the parentheses in the TM domain column refers to the number of TM domains the protein is predicted to have by SMART. [file peerj-02-264-s011.pdf]

B. hyodysenteriae gene	# ANK repeats	Signal Peptide	TM Domain
BHWA1_00072	19	✓	
BHWA1_00104	5		
BHWA1_00107	12		
BHWA1_00116	11	✓	
BHWA1_00208	7	✓	
BHWA1_00209	9	✓	
BHWA1_00220	4		
BHWA1_00221	14		
BHWA1_00248	10	✓	
BHWA1_00249	16	✓	
BHWA1_00295	5	✓	
BHWA1_00303	15		
BHWA1_00361	6	✓	
BHWA1_00390	2	✓	
BHWA1_00408	14		✓ (1)
BHWA1_00479	3		
BHWA1_00480	2	✓	
BHWA1_00481	5	✓	
BHWA1_00591	8	✓	
BHWA1_00593	10		✓ (1)
BHWA1_00594	5		
BHWA1_00595	2	✓	
BHWA1_00596	8	✓	
BHWA1_00684	6	✓	
BHWA1_00734	3		
BHWA1_00794	5	✓	
BHWA1_00854	9	✓	
BHWA1_00980	16		
BHWA1_00981	17		
BHWA1_01036	3	✓	
BHWA1_01055	4	✓	
BHWA1_01056	2		
BHWA1_01058	4	✓	
BHWA1_01059	4	✓	
BHWA1_01096	7		
BHWA1_01125	6	✓	
BHWA1_01126	7	✓	
BHWA1_01127	7	✓	
BHWA1_01128	2		
BHWA1_01195	3		✓ (3)
BHWA1_01259	3		✓ (1)
BHWA1_01425	4	✓	
BHWA1_01790	5		
BHWA1_01877	4		
BHWA1_01878	8	✓	
BHWA1_01895	9	✓	
BHWA1_02033	8	✓	
BHWA1_02034	8	✓	
BHWA1_02035	7	✓	
BHWA1_02162	15		
BHWA1_02195	4	✓	
BHWA1_02196	3	✓	
BHWA1_02197	3	✓	
BHWA1_02198	4	✓	
BHWA1_02271	6		
BHWA1_02343	2		
BHWA1_02414	7		
BHWA1_02441	3		
BHWA1_02448	4		
BHWA1_02450	7		
