# Supplementary material for: Ankyrin domains across the Tree of Life
Source: PeerJ. 2014 Feb 6;2:e264. doi: 10.7717/peerj.264 (PMC3932732; doi:10.7717/peerj.264)
Supplement: Supplemental Information 12 [file peerj-02-264-s012.pdf]

Genome	Ankryin proteins
Campylobacter concisus 13826	2
Campylobacter curvus 525.92	2
Campylobacter fetus subsp. fetus 82-40	2
Campylobacter hominis ATCC BAA-381	3
Campylobacter jejuni RM1221	2
Campylobacter jejuni subsp. doylei 269.97	2
Campylobacter jejuni subsp. jejuni 81-176	2
Campylobacter jejuni subsp. jejuni 81116	2
Campylobacter jejuni subsp. jejuni IA3902	3
Campylobacter jejuni subsp. jejuni ICDCCJ07001	2
Campylobacter jejuni subsp. jejuni M1	2
Campylobacter jejuni subsp. jejuni NCTC 11168 = ATCC 700819	3
Campylobacter jejuni subsp. jejuni S3	3
Campylobacter lari RM2100	2
Helicobacter acinonychis str. Sheeba	0
Helicobacter bizzozeronii CIII-1	0
Helicobacter cetorum MIT 00-7128	0
Helicobacter cetorum MIT 99-5656	0
Helicobacter cinaedi PAGU611	3
Helicobacter felis ATCC 49179	0
Helicobacter hepaticus ATCC 51449	13
Helicobacter mustelae 12198	2
Helicobacter pylori 2017	0
Helicobacter pylori 2018	0
Helicobacter pylori 26695	0
Helicobacter pylori 35A	0
Helicobacter pylori 51	0
Helicobacter pylori 52	0
Helicobacter pylori 83	0
Helicobacter pylori 908	0
Helicobacter pylori B38	0
Helicobacter pylori B8	0
Helicobacter pylori Cuz20	0
Helicobacter pylori ELS37	0
Helicobacter pylori F16	0
Helicobacter pylori F30	0
Helicobacter pylori F32	0
Helicobacter pylori F57	0
Helicobacter pylori G27	0
Helicobacter pylori Gambia94/24	0
Helicobacter pylori HPAG1	0
Helicobacter pylori HUP-B14	0
Helicobacter pylori India7	0
Helicobacter pylori J99	0
Helicobacter pylori Lithuania75	0
Helicobacter pylori P12	0
Helicobacter pylori PeCan18	0
Helicobacter pylori PeCan4	0
Helicobacter pylori Puno120	0
Helicobacter pylori Puno135	0
Helicobacter pylori Sat464	0
Helicobacter pylori Shi112	0
Helicobacter pylori Shi169	0
Helicobacter pylori Shi417	0
Helicobacter pylori Shi470	0
Helicobacter pylori SJM180	0
Helicobacter pylori SNT49	0
Helicobacter pylori SouthAfrica7	0
Helicobacter pylori v225d	0
Helicobacter pylori XZ274	0
