# Supplementary material for: Ankyrin domains across the Tree of Life
Source: PeerJ. 2014 Feb 6;2:e264. doi: 10.7717/peerj.264 (PMC3932732; doi:10.7717/peerj.264)
Supplement: Supplemental Information 14 — TM, transmembrane domain. The number in the parentheses in the TM domain column refers to the number of TM domains the protein is predicted to have by SMART. [file peerj-02-264-s014.pdf]

H. hepaticus gene	# ANK repeats	Signal Peptide	TM domain
HH0042	4		
HH0050	4		
HH0051	3		
HH0125	2		✓ (1)
HH0126	3		✓ (1)
HH0213	3		
HH0559	4	✓	
HH0814	3		
HH0925	4		
HH1187	7	✓	
HH1271	2	✓	
HH1376	3		
HH1405	3		
