# Supplementary material for: Ankyrin domains across the Tree of Life
Source: PeerJ. 2014 Feb 6;2:e264. doi: 10.7717/peerj.264 (PMC3932732; doi:10.7717/peerj.264)
Supplement: Supplemental Information 15 — TM, transmemebrane domain. The number in the parentheses in the TM domain column refers to the number of TM domains the protein is predicted to have by SMART. * Desti_0449 has a HPT (Histidine Phosphotransfer), a REC (cheY-homologous receiver) and a GGDEF(diguanylate cyclase) domain. **Desti_1504 has a Peptidase C14 (Caspase domain) domain. [file peerj-02-264-s015.pdf]

D. tiedjei gene	#Ank repeats	Signal peptide	TM domain	Other domain
Desti_0108	7			
Desti_0109	14			
Desti_0110	6			
Desti_0123	6	✓		
Desti_0423	2			
Desti_0449	4			✓ *
Desti_0581	2		✓ (1)	
Desti_0621	7	✓		
Desti_0634	13	✓		
Desti_1029	19	✓		
Desti_1504	12	✓		✓ **
Desti_1904	11	✓		
Desti_2412	9	✓	✓ (4)	
Desti_2485	5	✓		
Desti_2943	13	✓		
Desti_3186	10			
Desti_3335	15			
Desti_3392	3			
Desti_3481	10	✓		
Desti_3485	14	✓		
Desti_3962	5	✓		
Desti_3965	5			
Desti_4073	10			
Desti_4168	5			
Desti_4205	5			
Desti_4229	7	✓		
Desti_4389	3			
Desti_4485	4	✓		
Desti_4583	23			
Desti_4612	10			
Desti_4613	6			
Desti_4766	4	✓		
Desti_4853	9			
Desti_4969	8	✓		
Desti_5044	21			
Desti_5063	5			
Desti_5169	8			
Desti_5172	11	✓		
Desti_5175	7	✓		
Desti_5480	7			
Desti_5576	3			
Desti_5617	6	✓		
